# Help biocurators to maximize the reach of your data

**DOI:** 10.1371/journal.pbio.3002477

**Published:** 2024-01-25

**Authors:** Alexander Holmes, Denise Carvalho-Silva, Zbyslaw Sondka, Madiha Ahmed, Joanna Argasinska, Rachel Lyne, Amaia Sangrador-Vegas, Sari Ward

**Affiliations:** Wellcome Sanger Institute, Hinxton, Cambridge, United Kingdom

## Abstract

Scientific data is becoming increasingly complex, making data curation increasingly difficult. In this Perspective, a group of biocurators present some simple guidelines for data accessibility that will help to increase the reach of published studies.

## Teaser

Curated scientific databases catalogue and amplify research findings to maximize their reach. Authors should write their papers with this in mind, ensuring that data are accurate, easy to extract, and presented in standardized formats.

The academic paper is centuries old and is still the main method of discourse in research-focused environments. Whether as descriptions of novel findings or reviews of previous efforts, a paper and its citations are the threads from which theories and findings are woven from knowledge. However, the more we know, the greater the volume of publications. Where once a scientist of independent means could stay abreast of progress in most areas, there are now tens of thousands of papers published annually in any given field, and in total, PubMed currently holds over 36 million citations. It is now impossible for a single researcher to be familiar with even a modest percentage of these. This leads to a significant problem; how can data from a single paper contribute to a wider field when it may never be read by any more than a fraction of that field [1,2]?

Although the readership might appear to be limited, the data within have the potential to spread much further than authors may realize because of a particular type of reader—the biocurator. When a paper is curated, some or all of its data will be extracted, contextualized, and catalogued by data resources such as those at the Alliance of Genome Resources (AGR), EMBL-European Bioinformatics Institute (EMBL-EBI), or the US National Institutes of Health (NIH). These make it easy to find and view data, and, as they have many thousands of users, they operate as amplifiers and synthesizers of research to maximize the reach and impact of any single paper.

By curating a paper and integrating it within the wider data resource environment, the reach, impact, and value of its data are profoundly enhanced. To give an example, at the Catalogue of Somatic Mutations in Cancer (COSMIC), we created a resource of expert-curated somatic mutation information relating to human cancers from over 60,000 unique users that has contributed to thousands of publications. Beyond academia, the data we collate are routinely used in pharmaceutical research, diagnostic kit development, and to support clinical decisions throughout the world. Other resources have even greater reach and impact; a report commissioned by EMBL-EBI estimates that their services add billions of pounds of value annually to research impacts [3]. In the future, these resources are likely to increase further in value for sectors that utilize big data–dependent approaches.

However, to effectively empower such resources, the data within papers must be curatable; the data should be accurate, easy to extract, and presented in standardized formats. Unfortunately, this is not always the case, and all curators experience some consistent and longstanding problems across the biological literature that hinder curation. While the application of advances in artificial intelligence will advance the field, the problems we identify below will likely persist. Although some of these issues have been discussed in the literature [4–6], many tend to be discussed informally within the curation community and do not reach the general biology community. The main problems include the following:

1. **Not publishing the underlying data.** This is the most obvious problem and the easiest to rectify. Summary tables and figures are presented, but the underlying data are often missing. While you can turn a carrot into cake, you cannot turn a cake into a carrot. It is relatively easy to turn text and numbers into a nice figure, but to turn that figure back into raw data is often impossible. This could be solved quite simply by publishing all the underlying data.
2. **Inappropriate formatting.** A restaurant would not serve you a photo of the meal you ordered. If you have a spreadsheet, why would you save it as an image file? Not being able to copy and paste data or to clearly read it decreases the possibility of curation. Again, this can be solved simply by paying attention to proper formatting.
3. **Annotation and accessibility of data in external repositories.** The use of repositories is often recommended by funders and journals, and there are sound reasons for this: They increase trust and confidence in the quality of data, help align it with the FAIR principles, and increase the number of citations. However, authors and reviewers need to consider the accessibility and presentation of any submitted data. Being publicly available in principle and in practice are often not the same thing, as Douglas Adams wrote in relation to an important piece of planning permission: “It was on display in the bottom of a locked filing cabinet stuck in a disused lavatory with a sign on the door saying ‘Beware of the Leopard’” [7]. This is perhaps the biggest and most controversial problem for external repositories, given their popularity. Repositories are frequently inaccessible to curators due to access permissions, and there is a lack of sufficient control on the content and format of submitted data. Often, only raw data are included, particularly for genomic sequencing data, meaning that complex bioinformatic processing is required to recapitulate the summarized data in the original paper. This is often practically impossible for curators to do, as methodologies can be unclear or use bespoke and unavailable tools. There are also considerable risks that data are lost or made otherwise inaccessible when repositories undergo budgetary contractions and/or are retired [8–10]. If external repositories are used, then authors should ensure that processed data (such as vcf files) as well as raw data are uploaded and that access is not restricted or otherwise impaired.
4. **Third-party services restricting data.** This is a relatively new but growing problem. Have you ever bought a car only to find that you have to pay a lot more money simply to unlock some of its features? Some providers of sequencing services do not release all the data they generate back to researchers as standard; instead, researchers may only get a partial description of mutations, not the complete details. Communities, authors, and journals can solve this by establishing minimal datasets and standards, such as those that already underlie AGR resources, and we are pleased to note that such discussions are already happening elsewhere [11,12].
5. **Accuracy.** Occasional small errors in complex works are understandable, despite the best efforts of authors and reviewers to minimize these. Curators can help by correcting obvious mistakes. However, frequent small errors affect the quality of the work and will affect decisions to curate. Quality control tools could be developed for use prior to submission to help reduce this problem.

All the above are potentially rectifiable, but this leads to the ultimate problem: When asked, too many authors do not respond to requests to share their data despite this being a condition of publication and/or funding. Even when publishers mandate data sharing, requests are often ignored [13,14]. This represents a serious threat to the ability of data resources to extract data, as well as to the general credibility of research in general. How can we solve this problem?

Incentivizing the sharing of data requires the involvement of many stakeholders. Data resources could cite source publications in a way that counts towards a paper’s total citations. Some large funding agencies already insist on postpublication data sharing upon request (e.g., the NIH and UK Research and Innovation), and this should be expanded and enforced. Institutions could regard the failure to share data by authors as a notifiable offence. Journals could encourage curatable formats and robustly enforce data sharing commitments. Ultimately though, the responsibility will fall on authors as the creators and initial custodians of their data.

Modern scientific publishing can place requirements on authors that, while necessary, can be time consuming and complex to satisfy, and our suggestions will no doubt risk adding further complexity and frustration to the publication process. We are conscious of this and recognize that there are many different perspectives to consider other than our own. While it would be unreasonable to expect authors to write papers solely to our requirements, we think that the single most important thing any author can do is to place as much of their data as possible in simple plain text documents as supplemental data. If a summary table is presented in the main text, then the underlying data should be published as well. If a data table is presented, it should be available as a spreadsheet, not (just) as an image, pdf, or other nonextractable format. Making it pretty or excluding data for the sake of layout is not important; curators just want to curate your papers as best we can and for your benefit. By including all your data in simple formats, you make your paper curatable and you make it easy for us to promote and amplify your data, and who would not want that?

## Abbreviations

AGR: Alliance of Genome Resources
COSMIC: Catalogue of Somatic Mutations in Cancer
EMBL-EBI: EMBL-European Bioinformatics Institute
NIH: National Institutes of Health
